# Shifting tides in the emigration patterns of Canadian physicians to the United States: a cross-sectional secondary data analysis

**DOI:** 10.1186/s12913-016-1908-2

**Published:** 2016-12-01

**Authors:** Thomas R. Freeman, Stephen Petterson, Sean Finnegan, Andrew Bazemore

**Affiliations:** 1 Department of Family Medicine, Centre for Studies in Family Medicine, Western University, London, ON Canada; 2 The Robert Graham Center for Policy Research in Family Medicine and Primary Care, Washington, D.C, USA

**Keywords:** Physician migration, Medical human resources, Physician supply

## Abstract

**Background:**

The relative ease of movement of physicians across the Canada/US border has led to what is sometimes referred to as a ‘brain drain’ and previous analysis estimated that the equivalent of two graduating classes from Canadian medical schools were leaving to practice in the US each year. Both countries fill gaps in physician supply with international medical graduates (IMGs) so the movement of Canadian trained physicians to the US has international ramifications. Medical school enrolments have been increased on both sides of the border, yet there continues to be concerns about adequacy of physician human resources. This analysis was undertaken to re-examine the issue of Canadian physician migration to the US.

**Methods:**

We conducted a cross-sectional analysis of the 2015 American Medical Association (AMA) Masterfile to identify and locate any graduates of Canadian schools of medicine (CMGs) working in the United States in direct patient care. We reviewed annual reports of the Canadian Resident Matching Service (CaRMS); the Canadian Post-MD Education Registry (CAPER); and the Canadian Collaborative Centre for Physician Resources (C3PR).

**Results:**

Beginning in the early 1990s the number of CMGs locating in the U.S. reached an all-time high and then abruptly dropped off in 1995. CMGs are going to the US for post-graduate training in smaller numbers and, are less likely to remain than at any time since the 1970’s.

**Conclusions:**

This four decade retrospective found considerable variation in the migration pattern of CMGs to the US. CMGs’ decision to emigrate to the U.S. may be influenced by both ‘push’ and ‘pull’ factors. The relative strength of these factors changed and by 2004, more CMGs were returning from abroad than were leaving and the current outflow is negligible. This study supports the need for medical human resource planning to assume a long-term view taking into account national and international trends to avoid the rapid changes that were observed. These results are of importance to medical resource planning.

## Background

Canada and the United States share the longest international border in the world separating the second and third largest countries by area. Both countries have strong medical training systems and maintain harmonized medical education standards through the Liaison Committee for Medical Education (LCME). The 1988 Canada-US Free Trade Agreement (FTA) and the 1993 North American Free Trade Agreement (NAFTA), combined with common standards makes movement of physicians across the Canada-US border relatively easy. Canadian educated physicians may go the US for specialty training and then remain there to practice. Others may emigrate seeking more career opportunities and higher remuneration. American citizens may study medicine in Canada, then return to the US for further training and to establish practice.

**Fig. 1:**
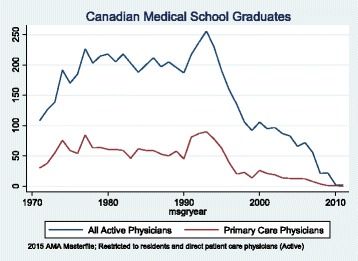
Trends in the Number of Canadian Medical School Graduates Practicing in the United States, by Year of Graduation from Medical School

**Fig. 2:**
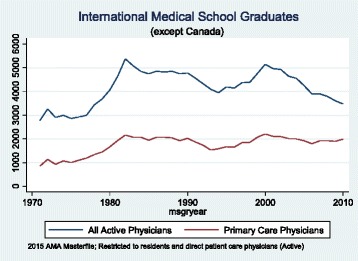
Trends in the Number of International Medical School Graduates Practicing in the United States, by Year of Graduation from Medical School

**Fig. 3:**
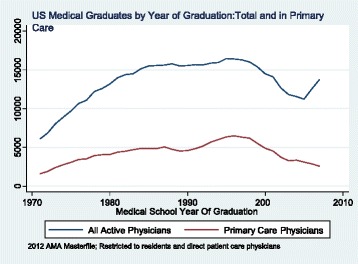
Trends in the Number of U. S. Medical School Graduates Practicing in Primary Care, by Year of Graduation from Medical School

**Fig. 4:**
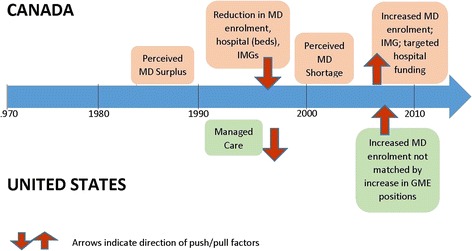
Timeline of Push/Pull Factors

Decisions about health human resources are the most important and costly ones made by leaders in healthcare [1]. While there are organizations in each country that make physician workforce projections at both national [2, 3] and sub-national [4, 5] levels, plans to address the barriers frequently become mired in political, jurisdictional and sectoral challenges [6, 7]. For example the National Health Workforce Commission established by the Accountable Care Act in the United States has not been funded or met [8]. Given the difficulty of estimating population healthcare needs, the length of a medical education and the complexity of jurisdictional control over postgraduate training, it is not surprising that real and perceived physician shortages and surpluses occur from time to time. It has been observed that Canadian educated physicians tend to move from less prosperous areas to more prosperous ones and that the physician-losing locales rely more on IMGs to deliver services [9]. Since Canadian physicians are more likely to move to the US, rather than the other way around, this north–south migration pattern has an effect on the number of IMGs needed to serve rural and underserviced areas and, thus, has implications beyond North America.

In Canada, a perceived surplus of physicians in the 1980s led to recommendations to limit the number of physicians by lowering medical school enrolment and reducing dependency on foreign trained physicians while also looking for other means of delivering health care [10]. As a result, there was a 10% reduction in medical school positions and immigrant physicians coming to Canada fell from a peak of 525 in 1993 to 243 in 1999 [11]. As a percentage of the Canadian physician workforce, IMGs declined from a high of 33% in the 1970s to 22.4% in 2007 [12]. Even before these measures were put in place, there were widespread reports of shortages in rural and small towns in Canada and by the 1990s, this became an issue in urban areas as well [13]. Concerns were raised about access to basic medical care and long waiting lists for elective surgery. The difficulties encountered in attaining access to health care had measurable, negative effects on population health [14, 15]. The movement of physicians from Canada to the US at this time was described as a ‘brain drain’ or ‘major exit ramp’ that contributed to physician shortages [16]. Phillips et al. [17], using data from the United States, shed light on this issue. They found that as of 2004, there were 8,162 Canadian educated physicians practicing in the US, 2,500 of them in primary care. It was estimated that between 1995 and 2004, 186 physicians from each year’s Canadian graduating class joined the US medical workforce and were more likely than US educated medical graduates to locate their practice in rural and underserviced areas. Further analysis suggested that this outflow of physicians was only a minor factor in the shortage of physicians in Canada [18].

The Canadian dialogue then shifted from physician surplus to physician shortages, and many of the initiatives that were aimed at reducing physician numbers in the 1990s were reversed. Between 1999 and 2013 medical school enrolment in Canada increased by more than 80% [19]. In the same time period, total postgraduate training positions increased 85% [20]. Efforts at primary care reform were undertaken in most provinces and, though differing in detail, were aimed at improving primary care physician income and providing infrastructure funding. There were initiatives to improve hospital funding intended to reduce surgical wait times for select procedures [21]. Restrictions on IMG immigration were reduced and their proportion of the physician pool increased [12].

At the same time, on the American side of the border, measures were also being taken to increase physician numbers. Between 2002 and 2017, medical school enrolment will have increased by 30%. This is projected to meet the expected requirements of an expanding and aging population [22]. However, to date, this expansion has not been matched by an increase in the number of Graduate Medical Education (GME) positions, creating a bottleneck in the training of American educated physicians and a barrier to Canadian and internationally trained physicians seeking to do postgraduate training in the US.

Both countries rely on international medical graduates (IMGs) to address shortfalls in health human resources [12, 23, 24]. Typically IMGs to Canada and the US have come from low and middle income nations creating physician shortages in donor countries [25], raising ethical concerns [26, 27]. Both countries are signatory to the World Health Organization Global Code on International Recruitment of Health Personnel [28] which recommends cross-border collaboration around data collection. Because of the national and international implications of the emigration pattern of Canadian physicians we decided to re-examine what changes, if any, have taken place in these patterns in years subsequent to Phillips et al. [17]. This information is pertinent to health human resource planning in both countries and the lessons learned may be of use to others involved in transnational physician workforce planning [29].

## Methods

We conducted a cross-sectional analysis of the 2015 American Medical Association (AMA) Masterfile to identify and locate any graduates of Canadian schools of medicine that were working in the United States in direct patient care. The AMA Masterfile aims to capture data on all physicians working in the United States, including name, demographics, origins, working addresses, type of practice, specialty type, location of medical school, and year of graduation.

We examined trends in the number of Canadian medical school students who graduated from 1971 to 2011 and were located in the United States. The 1971 start date was chosen to capture physicians prior to their retirement. We selected 2011 as the end date to account for the lag in the AMA Masterfile in updating address information of recent graduates of residency programs. This choice means that in more recent years we will not yet capture a small number of Canadian medical school graduates who also completed their residency training in Canada before emigrating to the United States. We limited our analysis to physicians either in practice or in a residency program in the United States as of 2015. We examined these trends for primary care physicians as well as all physicians. We also analyzed trends in the overall contribution of International Medical Graduates (IMGs) into the US physician workforce, as well as the rate of entry of US graduates into primary care.

We reviewed annual reports of the Canadian Resident Matching Service (CaRMS) which provides annual summaries of all medical graduates matched to Canadian postgraduate programs; the Canadian Post-MD Education Registry (CAPER) which captures data on postgraduate training and practice location following completion of training; and the Canadian Collaborative Centre for Physician Resources (C3PR) which provides statistical information on physician supply, migration and education in Canada.

We analyzed on a school by school basis, the contributions of individual Canadian medical schools to the US workforce.

The AMA Masterfile is available for purchase and its use is governed by a User-Customer Agreement. This study was fully compliant with the terms of this agreement including data security. No individual identifiers were utilized. The annual reports of the Canadian Resident Matching Service, the Canadian Post-MD Education Programs and the Canadian Collaborative Centre for Physician Resources are all freely available on the websites of those organizations.

## Results

Figure 1 displays the number of CMGs providing direct patient care in the US from 1971 to 2011. The upper line shows all active physicians and the lower line primary care physicians only. Graduates of Canadian medical schools emigrated to the US at a fairly steady rate in the two decades beginning in 1970. In the early 1990s there was a marked increase in this emigration pattern especially with specialist physicians, but clearly evident with primary care physicians as well. This pattern peaked in 1995. For primary care physicians and specialists there has been a rapid decline since 1995 to the point where emigration levels are negligible (Graph 1). Just 27 CMGs who graduated between 2009 and 2011 appear in the 2015 AMA Masterfile. It is possible that this number will increase slightly in the future as graduates who were also residents in Canada migrate to the United States (for 2000–2005 CMGs in the AMA Masterfile, about 10–15% also completed their residency in Canada).

Looking at IMGs (not including Canadian medical graduates) providing direct patient care in the US shows a somewhat different picture (Fig. 2). The number of IMGs in the US peaked in 1980 then began a gradual decline in 2001. This was the case for all physicians, but less dramatically for primary care physicians.

These trends reveal that there has been a decline in the total number of Canadian graduates and IMGs moving to the US, including primary care physicians. At the same time as the number of US medical graduates has increased, their interest in primary care specialties has declined as shown in Fig. 3. This contributes to a widening gap between specialist and primary care physicians in the medical workforce in that country.

Over 50% of Canadian medical school graduates working in the U.S. came from one of 4 medical schools: McGill University, University of Toronto, University of Manitoba and University of Alberta (Table 1).

**Table 1:** Location of Canadian medical school whose graduates were in US in 2015, by Medical School

Medical School	No (%) graduates ever in US		No (%) graduates currently in US		No (%) graduates currently in US in direct patient care	
McGill University	3603	21%	2873	25%	1674	23%
University of Toronto	2759	16%	1754	15%	1079	15%
University of Manitoba	1278	8%	880	8%	525	7%
University of Alberta	1226	7%	830	7%	553	8%
University of Western Ontario	1119	7%	743	6%	457	6%
Dalhousie University	986	6%	708	6%	458	6%
University of British Columbia	896	5%	544	5%	325	4%
University of Ottawa	895	5%	628	5%	388	5%
Queens University	805	5%	543	5%	328	5%
University of Montreal	668	4%	368	3%	209	3%
University of Saskatchewan	626	4%	431	4%	290	4%
McMaster University	551	3%	386	3%	266	4%
University of Calgary	487	3%	365	3%	273	4%
Laval University	478	3%	264	2%	135	2%
Memorial University	396	2%	295	3%	212	3%
University of Sherbrooke	209	1%	111	1%	77	1%
Total	16982	100%	11723	100%	7249	100%

Although Canadian graduates make up about 1% of the total physician workforce across the US, states such as North Dakota have proportions as high as 4.1%.

A review of the annual reports of the Canadian Resident Matching Service (CaRMS) [30] for each year from 2003 to 2012 revealed that the number of graduates of Canadian medical schools choosing to enter the residency match in the US (National Residency Matching Program) fell from 46 to 8. The Canadian Post-MD Education Registry (CAPER) maintains data on all postgraduate medical residents and fellows and issues an annual census including practice locations up to two years after completion of training. Examining the annual CAPER reports [31] for years 1995–2012, shows that those Canadian medical graduates remaining in the US 2 years after completing their training declined from 7.3 to 1.6% in that time period. In summary, fewer Canadian graduates are leaving for the US for postgraduate training and, of those that do, fewer are staying after completing training. These findings corroborate and may partially explain our observations.

The number of Canadian medical graduates practicing in direct patient care in the U.S. has dropped from a total of 8,162 in 2006 [17] to 6,709 in 2015 (Table 1) and few newer graduates are replacing them.

## Discussion

Decisions about health human resources are important and costly [1], yet are frequently made in the context of a lack of reliable data. This paper raises the importance of attending to physician migration across the Canada/US border as one variable that needs to be taken into account when making decisions about medical human resources in those countries.

The emigration of Canadian trained physicians to the US was a steady fixture between 1970 and 1990. This rate increased markedly in the 1990s raising alarms in Canada about a ‘brain drain’ and possible exacerbation of an alleged shortage of physicians [17], though Chan [18] later estimated that it contributed only 3% to the ‘perceived’ physician shortage. Our data show that this trend ended in the mid-1990s and actually reversed by 2004. We also found that fewer CMGs were applying to do postgraduate training in the United States, and of those who did, fewer remained there to practice.

One way of attempting to understand these trends is to view them through the ‘lens’ of push-pull factors [32]. Push factors are those that are considered to discourage physicians from remaining in a country and result in interest in leaving for what is perceived to be more favorable practice and living conditions. This might include issues of governance and health services management including hospital policies, lack of career opportunities, lack of funding for service and research and restrictions on income. Pull factors are those that are perceived as making another country a more attractive place to practice and live. These might include opportunities for further training, better living conditions, greater financial rewards, availability of practice positions and political and economic stability.

### Push/pull factors in the 1990s

There were a number of possible push factors identifiable in the early 1990s. Due to a perceived surplus of physicians in Canada, governments undertook policies to reduce medical school enrolment slots and the number of IMGs practicing in the country. During a time of economic stress, federal and provincial funding for health was cut and there were difficult negotiations over medical fee schedules between medical associations and provincial governments. Cost cutting was achieved through a reduction in hospital beds and health providers. As a result there was a general decline in confidence in the healthcare system [33]. Pull factors, at the same time in the US, included a shift toward managed care creating a need for physicians, especially those trained in a relatively cost conserving environment. Recruiters from the US were successful in attracting many Canadian graduates for practice and for specialty training. Many specialty physicians who went to the US for residency or fellowship training, remained there. A study of the 1989 class of all Canadian medical graduates found that 11.2% had relocated outside Canada, principally in the US [34, 35]. A study that compared all physicians who were certified in family medicine and who had been in practice for 8–10 years in 1993 and again in 1999 found that 6% had moved to the US in that time period [36]. Between 1990 and 1998, in Canada, the combination of push and pull factors as well as attrition due to retirements and deaths and population increases resulted in a decline of physicians per 100,000 population from 190 to 185 [13].

### Push/pull factors in the 2000s

In Canada, the first decade of the 21^st^ century saw increased medical school enrolment, more postgraduate residency positions and eased restrictions on IMG physicians entering the country. Push factors were reduced though efforts at health care reform including improved physician incomes and increased hospital funding to reduce surgical wait times. Between 1970 and 2007 provincial laws were changed allowing physicians in Canada to incorporate their medical practices [37] resulting in a lower tax burden and mitigating some of the income differential with US based physicians. On the pull side of the equation, the increase in medical school enrolment in the US, has not been matched by an increase in GME positions resulting in fewer positions for Canadian and IMG graduates wishing to pursue specialty training in the US. Further, there has been a 36% drop in non-immigrant visas in the two years following September 11, 2001 [38]. In Canada, by 2010, physician numbers had increased to 203 per 100,000 population [39].

This combination of policy changes, practice climate and economic factors leading to a reduction in push and pull factors may help explain the reversal of the physician ‘brain drain’ in Canada in the 1990s. As Canada trains more physicians and fewer emigrate to the US, it has been observed that the number of physicians in the country is increasing faster than the population [40] and concerns have been raised about the underemployment of some recently trained specialists [41]. The timeline of these push-pull factors is illustrated in Fig. 4.

It is important however, to recognize that ‘push-pull’ dynamics are fluid. Fallout from the recession of 2008 still affects Canadian provincial governments, setting the stage for difficult fee negotiations with provincial medical associations [42, 43]. Difficulty finding suitable employment for recently trained specialists, in part due to reduced hospital funding, all contribute to a potential increase in ‘push’ factors. On the other side of the border ‘pull’ factors are also changing. There are measures before the US Congress to correct the mismatch between medical school enrolment and GME numbers [44–46], which may attract more Canadian medical graduates seeking postgraduate training to that country. Taking into account projected demographic changes and the implementation of the Patient Protection and Accountable Care Act it has been estimated that the US will require a further 52,000 primary care physicians by 2025 [47]. This is occurring in the context of an observed decline in interest in primary care physicians in that country.

Traditionally, CMGs and IMGs have tended to fill primary care medical needs in rural and underserviced areas, but as the cohort of these physicians who were recruited to the US in the 1990s approach retirement, there will be an increased demand for their replacements. Under and unemployed specialty-trained physicians in Canada will once again be welcome in the US as well. It has been suggested that more effective team-based care, task substitution, and improvements in efficiency may mitigate some of the need for more physicians [48], but must take into account changing panel sizes [49, 50]. Increased activities of US 390 recruiters in Canada continue to be of concern [51, 52].

### Limitations

Inherent limitations of the AMA Physician Masterfile and in the cross-sectional design of our study may risk over-counting Canadian medical school graduates who train or practice in the United States and then return to Canada. Further, there is risk of undercounting physicians who have finished residency training but who are not yet counted in the physician workforce. There are limitations in measuring migration patterns, especially for non-respondents and in the years closest to graduation from residency training. Reliability appears to be poorest for the 3 to 5 years immediately after completion of residency training. Previous comparisons of AMA Physician Masterfile data suggested that this data lag may underestimate the number of Canadian trained physicians practicing in the United States by 10% or more [17]. It also prevents a clear picture of how migration has changed for three or more years. There is evidence of some lag time in accounting for physicians who have migrated. We believe that the evidence points to an underestimation of migration to the United States with a lag of 5 or more years.

## Conclusions

Providing adequate numbers of physicians to deliver medical care for the Canadian and US population requires consideration of many variables [51]. Physician migration across the Canada/US border is only one of them, but requires further understanding.

Over the past four decades there has been considerable fluctuation in the emigration pattern of Canadian trained physicians to the US with an unprecedented decline since 1995. ‘Push’ and ‘pull’ factors may help explain these changes. Emerging policy developments on both sides of the Canada/US border may have a substantial impact on physician migration and further understanding of this dynamic is needed for planning. As both countries rely on IMGs to fill human resource gaps there are implications beyond North America. Both Canada and the US are signatories to the World Health Organization Global Code on International Recruitment of Health Personnel [28] which stipulates that member nations “…should strive, to the extent possible, to create a sustainable health workforce and work towards establishing effective health workforce planning, education and training, and retention strategies that will reduce their need to recruit migrant health personnel.” [Article 3.6]. Presently there is no infrastructure to support the analysis of the dynamics of physician workforce across North America, as exists in Europe [53]. The present study takes the first step in the recommendation that countries collaborate on cross border data collection for effective physician resource planning and to avoid the rapid changes seen here.

## Abbreviations

AMA: American Medical Association
C3PR: Canadian collaborative centre for physician resources
CMGs: Canadian medical graduates
CAPER: Canadian Post-MD Education Registry
CaRMS: Canadian resident matching service
IMG: International medical graduate
OECD: Organization for economic co-operation and development
US: United States
WHO: World Health Organization
